# Cellular and Transcriptional Responses of *Crassostrea gigas* Hemocytes Exposed *in Vitro *to Brevetoxin (PbTx-2)

**DOI:** 10.3390/md10030583

**Published:** 2012-03-05

**Authors:** Danielle F. Mello, Eliza S. de Oliveira, Renato C. Vieira, Erik Simoes, Rafael Trevisan, Alcir Luiz Dafre, Margherita Anna Barracco

**Affiliations:** 1 Laboratory of Immunology Applied to Aquaculture, Department of Cell Biology, Embryology and Genetics, Biological Sciences Center, Federal University of Santa Catarina, 88040-900, Florianópolis, SC, Brazil; Email: danifmello@gmail.com (D.F.M.); elizaoliveira91@gmail.com (E.S.O.); renatocamposvieira@gmail.com (R.C.V.); eriksimoess@yahoo.com.br (E.S.); 2 Laboratory of Cell Defense, Department of Biochemistry, Biological Sciences Center, Federal University of Santa Catarina, 88040-900, Florianópolis, SC, Brazil; Email: rafael.trevisan@gmail.com (R.T.); alcir@ccb.ufsc.br (A.L.D.)

**Keywords:** brevetoxin, hemocytes, bivalves, gene expression, immune, antioxidant and detoxification systems

## Abstract

Hemocytes mediate a series of immune reactions essential for bivalve survival in the environment, however, the impact of harmful algal species and their associated phycotoxins upon bivalve immune system is under debate. To better understand the possible toxic effects of these toxins, *Crassostrea gigas* hemocytes were exposed to brevetoxin (PbTx-2). Hemocyte viability, monitored through the neutral red retention and MTT reduction assays, and apoptosis (Hoechst staining) remained unchanged during 12 h of exposure to PbTx-2 in concentrations up to 1000 µg/L. Despite cell viability and apoptosis remained stable, hemocytes incubated for 4 h with 1000 µg/L of PbTx-2 revealed higher expression levels of *Hsp70* (*p* < 0.01) and *CYP356A1* (*p *< 0.05) transcripts and a tendency to increase *FABP* expression, as evaluated by Real-Time quantitative PCR. The expression of other studied genes (*BPI*, *IL-17*, *GSTO*, *EcSOD*, *Prx6*, *SOD* and *GPx*) remained unchanged. The results suggest that the absence of cytotoxic effects of PbTx-2 in *Crassostrea gigas* hemocytes, even at high concentrations, allow early defense responses to be produced by activating protective mechanisms associated to detoxification (*CYP356A1* and possibly *FABP*) and stress (*Hsp70*), but not to immune or to antioxidant (*BPI*, *IL-17*, *EcSOD*, *Prx6*, *GPx* and *SOD*) related genes.

## 1. Introduction

Brevetoxins represent a group of polyether compounds known to cause neurotoxic shellfish poisoning (NSP) through the consumption of brevetoxin-containing shellfish. They are produced mainly by the marine dinoflagellate *Karena brevis*, but recently brevetoxin-like compounds were also found in other microalgae species such as *Chatonella marina*, *Chatonella antiqua*, *Fibrocapsa japonica* and *Heterosigma akashiwo* [1]. Besides the threats to humans, blooms of these neurotoxin-producing species occur worldwide, causing massive fish kills along with bird and marine mammal mortalities through the direct exposure to these blooms, or indirectly by the food web [2]. *K. brevis*, mainly produces two brevetoxins types, with PbTx-1 being the most potent and PbTx-2 being the most abundant [3]. Bivalves accumulate and metabolize these toxins, producing oxidized, reduced, hydrolized and conjugated metabolites that vary in their toxic potency [4,5].

Although shellfish appear to be only toxin vectors unaffected by harmful algal blooms (HABs), some behavioral, physiological and cellular responses of bivalves to harmful algae have already been described, along with mortality events [2,6]. Responses of bivalves associated to brevetoxin or brevetoxin-producing algae include reduced clearance rates [7], impairment of larval survival and development [8,9] and increases in lysosomal destabilization in oyster hepatopancreas [10,11]. Most recently, the HAB species *H. akashiwo*, described to produce brevetoxin-like compounds [12], affected significantly bivalve immune cells viability and phagocytosis *in vitro* [13].

The study of the effects of harmful algae upon bivalve immune system has recently become an area of interest of researchers. Various publications have demonstrated that hemocytes and immune parameters can be activated or modulated by several toxic microalgae species [13,14,15,16,17,18,19,20,21,22,23,24]. However, to our knowledge, analysis of the effects of a purified brevetoxin upon the immune system of bivalves has never been assessed, especially at the gene expression level.

Bivalve innate immune responses are mediated by hemocytes. They are involved in cellular immune reactions, such as phagocytosis of invading microorganisms, formation of nodules and capsules surrounding pathogens and their subsequent degradation through the release of host defense molecules [25]. Moreover, circulating hemocytes are able to migrate from the hemolymph to connective tissues and promote localized responses following injury or microorganism invasion.

The Pacific oyster *Crassostrea gigas* (Thunberg, 1793) is a bivalve mollusc belonging to the Ostreidae family (Mollusca, Bivalvia), with a global distribution and probably accounts for the highest annual production of any freshwater or marine organism [26]. This species has a reasonable number of cDNA sequences deposited in GenBank, which permits the study of several environmental stressors at the gene expression level.

The objective of this study was to evaluate the *in vitro* effects of the purified brevetoxin PbTx-2 over *C. gigas* hemocyte viability, apoptosis and gene expression.

## 2. Results

### 2.1. Cellular Parameters

**Figure 1:**
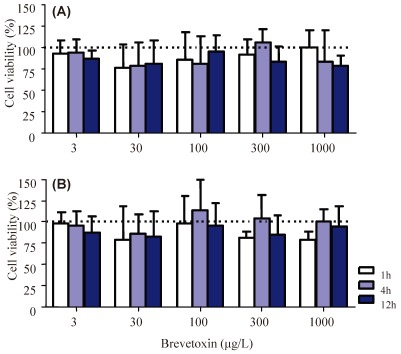
Viability of *Crassostrea gigas* hemocytes evaluated through the neutral red (NR) retention (**A**) and 3-(4,5-dimethylthiazol-2-yl)-2,5-diphenyltetrazolium bromide (MTT) reduction (**B**) assays after exposure to different brevetoxin (PbTx-2) concentrations. Bars represent mean + standard deviation. The sample size was 3–4, performed in independent experiments. The range of individuals used varied from 5 to 30 oysters per pool, depending on the number of treatment groups analyzed in each experiment. No significant differences were obtained in the analysis of variance.

In order to investigate whether PbTx-2 could cause hemocyte toxicity, cell viability assays were performed using the MTT and NR methods. No significant differences were observed in the viability of *C. gigas* hemocytes after incubation with PbTx-2 concentrations ranging from 3 to 1000 µg/L for 1, 4 and 12 h, according to either viability assay (Figure 1). 

Apoptotic hemocytes (Supplementary Figure S1) were also quantified using Hoechst staining after cell exposure to the highest concentrations of PbTx-2 for 12 h. The percentage of hemocytes presenting characteristic altered nuclei remained unchanged after incubation with 300 (4.7 ± 3.4%) or 1000 (3.2 ± 1.8%) µg/L of PbTx-2 when compared to the control group (3.1 ± 1.6%).

### 2.2. Gene Expression Levels

The gene expression analysis was evaluated after hemocytes were incubated during 4 h with the highest concentrations of PbTx-2 analyzed in the viability assays, in view of the fact that no cytotoxic effects were observed. Previous studies have demonstrated that a 4 h incubation period is sufficient to induce gene expression and obtain early molecular responses in hemocytes [27,28]. The immune related genes selected are involved in pathogen recognition (*EcSOD*) and elimination (*BPI*) and cell signaling (*IL-17* and possibly *Prx6*). Also other genes associated to the antioxidant system, stress and detoxification were selected (*GPx*, *SOD*, *Hsp70*, *GSTO*, *CYP356A1* and *FABP*), to span various branches of oyster defense systems. In previous studies the genes *GSTO*, *CYP356A1* and *FABP* were significantly induced following *C. gigas* exposure to sewage [29]. Prx6 protein levels decreased after *in vivo* exposure to zinc in gills of the brown mussel *Perna perna* [30] and also its gene expression was modulated in oysters selected for disease resistance [31] and in oysters sampled from contaminated estuaries [32]. GPx and SOD are major antioxidant players that can be modulated by different xenobiotics [33] and their gene expression analysis could demonstrate any up or downregulation of the antioxidant system.

**Figure 2:**
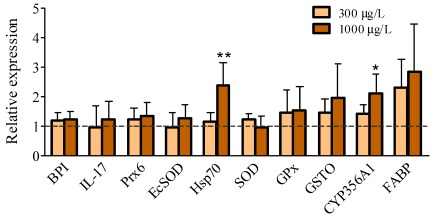
Relative expression of the 10 gene transcripts (normalized to *GAPDH*) in *Crassostrea gigas* hemocytes exposed for 4 h to 300 or 1000 µg/L of PbTx-2. The dashed line represents the mean value of the control groups (vehicle) for each gene. Bars represent mean + standard deviation. The sample size was comprised of 4 pools of 5–9 animals. * (*p *< 0.05) and ** (*p *< 0.01) represent significant differences between each gene compared to the corresponding control group.

In the present study, *C. gigas* hemocytes presented a more than 2-fold increase (*p *< 0.05) in the transcript levels of genes encoding *Hsp70* and *CYP356A1*, but only when incubated with the highest concentration of PbTx-2 (1000 µg/L) (Figure 2). All of the other transcripts analyzed, however, showed similar expression profiles between the control and treated groups, which included *BPI*, *IL-17*, *FABP*, *EcSOD*, *Prx6*, *GPx*, *SOD* and *GSTO* (Figure 2).

## 3. Discussion

Although many studies have assessed the effects of harmful algae and their toxins upon the immune system of bivalves, the biological interactions between them are still very poorly understood, especially at the molecular level. Therefore, in this study we evaluated *C. gigas* hemocyte responses to purified PbTx-2 *in vitro*. The objective of this work was an initial screening of cellular and transcriptional effects of a purified ficotoxin upon bivalve immune cells. Although other brevetoxin metabolites were already detected in oysters tissues [4,5], PbTx-2 was chosen since it is the most abundant brevetoxin produced by *Karenia brevis* during natural blooms [3].

Hemocytes may have multiple opportunities to become in direct contact with toxins during HABs events. Firstly, in the pallial cavity where hemocytes can be found protecting host from potential pathogens even before invasion [34,35]. Secondly, after filter-feeding and digestion of the harmful algae, when intracellular toxins can be released and end up in animal tissues and semi-open vascular system where hemocytes are plentiful [36]. Ford *et al.* [36] discussed that hemocytes can migrate through the intestine epithelium, phagocyte partially digested algae and return to circulation in the hemolymph. Moreover, Franchini *et al.* [37] showed a great quantity of another phycotoxin, the yessotoxin, in the cytoplasm of mussel hemocytes through immunolocalization. Therefore, there are multiple opportunities for *in vivo* toxins-hemocyte encounters.

The absence of effects of a purified brevetoxin upon hemocyte viability, even in the highest concentration and time exposure tested, was unexpected. Also, no changes in the percentage of apoptotic hemocytes were observed, which supports the idea of an absence of gross toxic effects of brevetoxin upon hemocytes. On the other hand, these results may not be so surprising, since the toxic mechanism of this toxin is through the blocking of voltage-gated sodium channels of excitable cells [1]. Furthermore, these results are consistent with other studies, which observed no toxic effects in bivalve hemocytes exposed *in vitro* and *in vivo* to microalgal species that produce saxitoxin (STX), a neurotoxin which has a mechanism of action related to brevetoxin [36,38]. It is important to note that the use of PbTx-2 *in vitro* does not take in account the metabolization that could take place *in vivo*, producing metabolites that could be toxic to oyster immune cells.

The lack of effects of PbTx-2 in the cellular parameters tested, could probably also be explained by the induction of genes related to protective mechanisms. In this study, the results of the gene expression analysis showed an increase in the transcriptional levels of *Hsp70* and *CYP356A1*.

The induction of chaperones, such as Hsp70, in response to thermal stress has already been demonstrated in many organisms, including oysters [39,40,41,42,43]. Additionally, an increase in the expression of heat shock proteins was also reported in a variety of bivalves species exposed to toxic compounds or contaminated sites [44,45,46,47,48,49,50]. In bivalves, increases in the expression of Hsp70 were also associated with infections [46,51,52,53]. These studies, together with the result obtained in the present study, support the role of *Hsp70* as a response to stress in general and not only to thermal shock [54,55]. Furthermore, the upregulation of Hsp70 could recurrently be associated to increases in animal resistance to stressors, including toxic compounds [46,55,56].

The cytochrome P450 (CYP) family is characterized as one of the major phase I-type classes of detoxification enzymes found in living organisms, including the aquatic organisms. These enzymes metabolize a wide variety of substrates, such as fatty acids, hormones and xenobiotics [57]. According to Toledo-Silva *et al.* [58], the cytochrome isoform *CYP356A1*, has great similarity to the CYP17 family, which is associated with the metabolism of steroids, but also a lower similarity to the CYP1 family, which comprises CYP1A, commonly associated with the metabolism of polycyclic aromatic hydrocarbons. Despite the debate on the existence o CYP-dependent phase I of biotransformation, CYP356A1 has the potential to participate in the brevetoxins-metabolism. Bivalves accumulate and extensively metabolize these toxins, producing oxidized, reduced, hydrolized and cysteine conjugated metabolites [4,5]. To our knowledge, the metabolizing pathways of brevetoxins have not been described yet in these organisms. On the other hand, PbTx-2 metabolization associated to cytochrome P450 activity was confirmed in human, rat and fish CYPs [59,60,61]. Within metabolites described in these studies, some corresponded to metabolites identified in oyster tissues, e.g., PbTx-3 and PbTx-9 [62,63]. Therefore, the increase in the transcriptional level of a CYP isoform found in the present study opens the opportunity for testing this enzyme in the biotransformation of PbTx-2.

GST is key enzyme of phase II biotransformation pathway, indicated to be similar between aquatic invertebrates and other vertebrates, is responsible for the conjugation of glutathione (GSH) to electrophilic compounds, increasing its hydrophilicity and allowing the elimination of these conjugates into the extracellular environment [64]. Previous studies have suggested that rat and fish GSTs could be associated to the metabolization of PbTx-2 [60,61]. Moreover, brevetoxin conjugated to GSH and its corresponding dipeptides were found in oyster tissues [62]. Unexpectedly, the transcript levels of the gene encoding GST omega (*GSTO*) did not show significant differences between hemocytes exposed to the highest concentration of PbTx-2. It is important to note that the gene expression of only one GST isoform was evaluated, therefore it is possible that other isoforms could be involved in metabolizing PbTx-2 and be up-regulated. In addition, according to Livingstone *et al.* [64], Phase II enzymes in aquatic invertebrates, such as GST, are generally much less responsive to organic xenobiotics exposure than CYPs.

Another protein, possibly involved in detoxification pathways is FABP, a small cytosolic molecule with affinity for hydrophobic compounds. Although its functions are not yet fully understood, it is believed that FABP is involved in the uptake and transport of fatty acids [65]. However, this molecule can also be associated with xenobiotic metabolism since Velkov *et al.* [66] reported that the *FABP* has high affinity for lipophilic drugs and Medeiros *et al.* [29] observed an induction of its gene in oysters exposed to domestic sewage. In the present study, the level of *FABP* transcripts were increased ~2.5 in hemocytes exposed to both concentrations of PbTx-2, when compared to the control group, but significance was not reached. This result suggests that FABP may be involved in the metabolism of PbTx-2 in hemocytes, which remains to be confirmed in future studies.

None of the studied genes encoding proteins associated to the immune system were modulated. Among some immune effector molecules already described in *C. gigas*, we can cite (1) BPI, which increases membrane permeability leading to bacterial cell extravasation and can be induced in hemocytes of oysters by challenge with bacteria [67]; (2) IL-17, a cytokine similar to the IL-17 of vertebrates, which its first report in an invertebrate was in *C. gigas* and can also be induced in hemocytes after bacterial challenge [68]; (3) EcSOD, a protein with superoxide dismutase activity, that also has the property to bind to lipopolysaccharide (LPS) present on the surface of Gram (−) and is believed to act as an opsonin by binding to integrins of the membranes of hemocytes [69], thus enhancing recognition and elimination of bacteria through phagocytosis; and (4) Prx6, an enzyme implicated in the antioxidant defenses, with only one cysteine residue in its active site, involved in the metabolism of peroxides [32]. Recent studies regarding vertebrates and invertebrates (helminths) suggest that the peroxiredoxins can assume an important role in the immune system, mediating the activation of defense cells [70]. Along with Prx6, other proteins associated to antioxidant defenses did not show any significant modulations in their transcript levels, such as SOD and GPx. SOD enzyme is a metalloprotein responsible for the dismutation of superoxide (O_2_^•−^) to hydrogen peroxide (H_2_O_2_) and was identified in *C. gigas* by Boutet *et al.* [45], whereas GPx is a selenium protein that has peroxidase activity against H_2_O_2_ and organic peroxides, described in the Pacific oyster by Jo *et al.* [71]. 

## 4. Experimental Section

### 4.1. Animals

Adult oysters of *C. gigas* (Thunberg, 1793) species were obtained from a mariculture farm located in the southern bay of Florianópolis, Santa Catarina, southern Brazil (27°38′56″S and 48°32′31″W). Oysters were acclimated for a minimum period of 10 days and kept in filtered seawater (FSW-1 µm) at 18–20 °C, before their use in experiments. During this period, the oysters were fed with the microalgae *Tetraselmis *sp. The experiments here presented are in accordance to the Federal University of Santa Catarina ethic committee on the animal use (CEUA).

### 4.2. Hemolymph Extraction and Hemocyte Incubation with Brevetoxin (PbTx-2)

The oyster hemolymph (4–5 pools of 5–30 animals) was extracted from the adductor muscle by inserting a 0.8 × 30 mm needle attached to a 1 mL syringe, after making a small hole in the valves of the animals close to the muscle. The hemolymph was maintained on ice until use. Immediately after hemolymph collection, the total number of hemocytes was determined using a Neubauer hemocyte chamber. In order to collect hemocytes, hemolymph was centrifuged (600 g, 10 min, 4 °C) and the hemocyte pellet was resuspended in FSW (0.22 µm) to obtain a concentration of 1 × 10^6^ cells/mL.

For the viability assays, hemocyte suspensions (1 × 10^6^ cells/mL) were incubated with 3, 30, 100, 300 and 1000 µg/L of purified PbTx-2 (World Ocean Solutions, Wilmington, EUA), previously solubilized in dimethyl-sulfoxide (DMSO - at a maximal final concentration of 0.05%) for 1, 4 and 12 h at 18 °C, in the dark. For the control groups, hemocytes were incubated with FSW containing the corresponding DMSO concentration (vehicle). For the quantification of apoptotic hemocytes, cells were incubated for 12 h with 300 or 1000 µg/L of PbTx-2 or the corresponding vehicle, while for the gene expression analysis the incubation period was only for 4 h.

### 4.3. Cellular Viability Assays and Apoptosis

After treatments, oyster hemocytes were used to determine their viability, using the 3-(4,5-dimethylthiazol-2-yl)-2,5-diphenyltetrazolium bromide (MTT) or neutral red (NR) assays following established methods as previously employed by Trevisan *et al.* [72]. A total of 6 × 10^5^ (MTT) and 3 × 10^5^ (NR) cells were used for each assay and conducted in triplicate. The hemolymph was pooled to obtain the number of cells necessary for the analysis, with a total of 3–4 pools (*N* = 3–4). The range of individuals used varied from 5 to 30 oysters per pool, depending on the number of treatment groups analyzed in each experiment.

The percentage of potentially apoptotic hemocytes was determined by using Hoechst 33258 staining (Sigma-Aldrich, São Paulo, Brazil). Fixed hemocyte monolayers (*N* = 3 pools of 5–7 animals) were immersed in McIlvane buffer (0.1 M citric acid, 0.4 M disodium hydrogen phosphate, pH 5.5) for 5 min and then treated with a Hoechst solution in McIlvane buffer (1:1500) for 5 min. The slides were then mounted with cover slips and observed under a fluorescence microscope (excitation of 365 nm). The percentage of apoptotic cells was estimated by examining 300 cells per group and counting morphologically altered nuclei characteristic of apoptotic cells (Supplementary Figure S1). All cell types were represented in the samples (both hyaline and granular hemocytes were observed) although it should be taken in account that hyaline hemocytes could be less represented since they may be less adherent to glass slides. 

### 4.4. Extraction of Total RNA and cDNA Synthesis

At the end of the incubation, samples (*N* = 4 pools of 5–9 animals) were centrifuged (1000 g, 10 min, 4 °C) and cells resuspended in RNALater^®^ (Invitrogen, São Paulo, Brazil) and stored until use. The RNA extraction from hemocytes was performed using TRIzol^®^ reagent (Invitrogen, São Paulo, Brazil). Samples were then treated with DNase I (Fermentas, São Paulo, Brazil) and RNA concentration and purity were assessed spectrophotometrically (NanoVue, GE Healthcare, Uppsala, Sweden). For cDNA synthesis, only RNA samples with absorbance ratio (A260/A280) between 1.8 and 2.1 were used. The cDNA synthesis was performed on 250 ng of total RNA using 0.5 µg of oligo (dT)_18_ primer (Fermentas, São Paulo, Brazil), 1 U of RevertAid^®^ Reverse Transcriptase (Fermentas, São Paulo, Brazil) and 20 U of RiboLock^TM^ RNase Inhibitor (Fermentas, São Paulo, Brazil).

### 4.5. Real-Time Quantitative PCR (qPCR)

cDNAs were used for qPCR analysis in order to determine the relative expression of mRNA coding ten proteins: bactericidal/permeability increasing protein (BPI), interleukin-17 (IL-17), extracellular superoxide dismutase (EcSOD), peroxiredoxin 6 (Prx6), heat shock protein (Hsp70), glutathione peroxidase (GPx), superoxide dismutase (SOD), glutathione S-transferase omega (GSTO), fatty acid binding protein (FABP) and a member of the cytochrome P450 family (CYP356A1). Primers (Table 1) that weren’t used in previous works were designed from specific sequences of *C. gigas* deposited in GenBank, with the following characteristics: size from 18–20 bp, Tm between 59–61 °C, GC content between 40–60% and amplicon size between 90–200 bp. The sequences and the GenBank accession numbers [73] are listed in Table 1. Primers efficiency was tested using the standard curve method. For this purpose, a serial dilution (1:3, 1:7, 1:15, 1:31, 1:63) was made from a single cDNA sample consisted of a pool of all cDNAs from the different treatments. Only primers that showed efficiencies between 1.8 and 2.2 were used. The qPCR analysis was performed in 96-well microplates, in duplicate, using the ABI 7900HT thermocycler (Applied Biosystems, São Paulo, Brazil), with a total reaction volume of 10 µL. Each reaction had 5 µL of Maxima SYBR^®^ Green/ROX qPCR Master Mix 2X (Fermentas, São Paulo, Brazil), 0.3 mM of each primer and 1 µL of each diluted cDNA (1:4). Amplification conditions were 95 °C for 10 min followed by 40 cycles of 95 °C for 15 s and 60 °C for 60 s. The specificity of the qPCR product was analyzed by a dissociation curve performed after amplification, observing a single peak at the expected Tm. The results were expressed as relative expression of transcripts normalized by the reference gene glyceraldehyde 3-phosphate dehydrogenase (*GAPDH*), using the 2^−(ΔΔct)^ method [74].

**Table 1:** Gene names and primer sequences used for Real-Time Quantitative PCR (qPCR) and GenBank accession numbers.

Gene Name	Primers	Sequence 5'-3'	GenBank
*Glyceraldehyde 3-phosphate dehydrogenase*	CgGAPDH-Fw	GCTGTGACACCATTGGAGAA	AJ544886.1
	CgGAPDH-Rv	ACCAATGACGCAACAAGCGA	
*Peroxiredoxin 6*	CgPrx6-Fw	GAGCCAGAGTTCAAGAAGAG	AM265552.1
	CgPrx6-Rv	TGCATTGTCCTTTTCGGCTG	
*Interleukin-17*	CgIL17-Fw	ACTGAGGCTCGATGCAAGTG	EF190193.1
	CgIL17-Rv	AGCCTTCTTGCTTCATGTGG	
*Bactericidal/permeability*	CgBPI-Fw	GATAGAAATAGGAATGGACGG	HM992925.1
*increasing protein*	CgBPI-Rv	GTTATAGATCCACGCTGCTCC	
*Heat shock protein 70*	CgHsp70-Fw	TCATCAAGTGGATGGACCAG	AB122063.1
	CgHsp70-Rv	CATTCCTCCAGGCATGCCA	
*Extracellular superoxide dismutase*	CgEcSOD-Fw	GCTGTGACACCATTGGAGAA	DQ010420.1
	CgEcSOD-Rv	ACCAATGACGCAACAAGCGA	
*Glutathione S-transferase omega*	CgGSTO-Fw	TGATGAGTTCACCACCGCAA	AJ557141.1
	CgGSTO-Rv	TTCAAACCATGGCCACAGCA	
*Cytochrome P450 isoform 356A1*	CgCYP3561A-Fw	ATGAAACCCGCGAAACCAGA	EF645271.1
	CgCYP3561A-Rv	TAAATTCGGCTTCACGCCCT	
*Superoxide dismutase*	CgSOD-Fw	TCAACAAAGAGCATGGCGTC	AJ496219.1
	CgSOD-Rv	TTTCCGGTCGTCTTACTGAG	
*Glutathione peroxidase*	CgGPx-Fw	TCAAGATCCGAGATGTCGTC	EF692639.1
	CgGPx-Rv	ACTCGGTTTCCAGACATGAG	
*Fatty acid binding protein*	CgFABP-Fw	GTTTGAGGGAAACTGGGAATGC	EU069496
	CgFABP-Rv	TCCGTCGGAATATGTCAGTTTAGC	

### 4.6. Statistical Analysis

The results of the viability assays were compared by two-way ANOVA, while qPCR results and quantification of apoptotic hemocytes were compared by one-way ANOVA, both followed by Tukey’s *post hoc* test for comparison of means. For the quantification of apoptotic hemocytes (percentages), the data were arcsin transformed. The results were considered significant at *p *< 0.05. Statistical analysis was performed using Statistica [75].

## 5. Conclusions

*In vitro* studies have many advantages, such as feasibility, control of variables, rapid results for screening experiments, lower number of animals and reproducibility. Our experiments showed that *Crassostrea gigas *hemocytes fully retained viability and did not undergo apoptosis during the first 12 h in the presence of PbTx-2, and that, apparently, PbTx-2 triggered defense mechanisms including activation of stress (*Hsp70*) and detoxification related genes (*CYP356A1* and possibly *FABP*), suggesting a specific response. However, further studies both *in vitro* and *in vivo* are necessary to confirm the real effects of brevetoxins upon the immune system of cultivated bivalves or wild stocks, particularly at the gene expression level.

## Supplementary Files

Supplementary File 1: PDF-Document (PDF, 44 KB)
