# Supplementary material for: Cellular and Transcriptional Responses of Crassostrea gigas Hemocytes Exposed in Vitro to Brevetoxin (PbTx-2)
Source: Mar Drugs. 2012 Mar 5;10(3):583–97. doi: 10.3390/md10030583 (PMC3347016; doi:10.3390/md10030583)

Supplmentary Material

Figure S1. Micrographs showing nuclear alterations suggesting apoptosis in hemocytes of *Crassostrea gigas* using Hoechst staining. (**A**) and (**B**) = normal nuclei; (**C**) and (**D**) = altered nuclei characteristic of apoptotic cells.

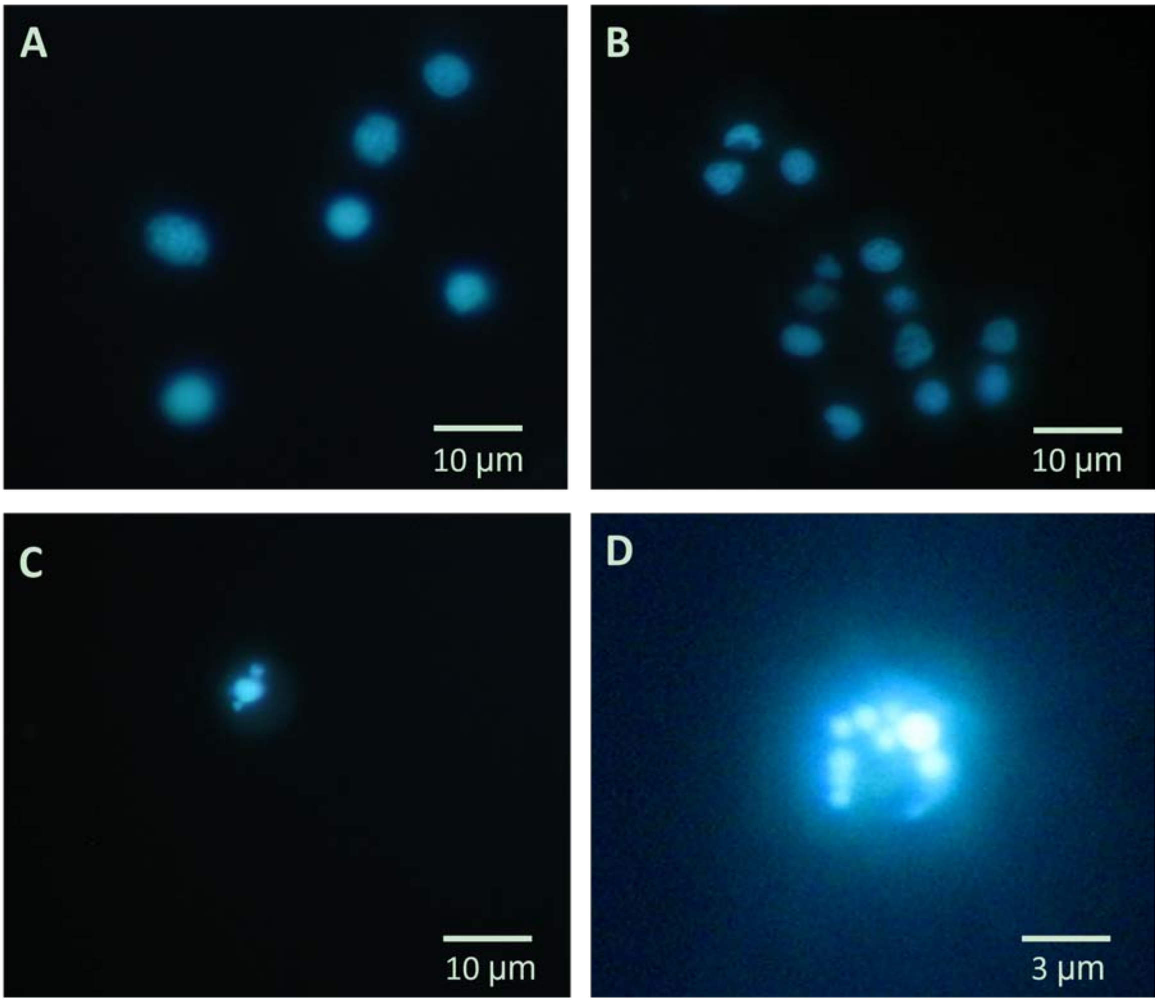

Supplement: Supplementary File 1: — PDF-Document (PDF, 44 KB) [file marinedrugs-10-00583-s001.pdf]
